# Unmanned Surface Vehicle Thruster Fault Diagnosis via Vibration Signal Wavelet Transform and Vision Transformer under Varying Rotational Speed Conditions

**DOI:** 10.3390/s24051697

**Published:** 2024-03-06

**Authors:** Hyunjoon Cho, Jung-Hyeun Park, Ki-Beom Choo, Myungjun Kim, Dae-Hyeong Ji, Hyeung-Sik Choi

**Affiliations:** 1 Department of Mechanical Engineering, Korea Maritime & Ocean University, Busan 49112, Republic of Korea; jooninkmou@kmou.ac.kr (H.C.); skrgus3020@g.kmou.ac.kr (J.-H.P.); 2 Interdisciplinary Major of Ocean Renewable Energy Engineering, Korea Maritime and Ocean University, Busan 49112, Republic of Korea; 3 Advanced-Intelligent Ship Research Division, Korea Research Institute of Ship & Ocean Engineering, Daejeon 34103, Republic of Korea; kbchoo@kriso.re.kr; 4 Maritime R&D Center, LIG NEX1 Co., Ltd., Seongnam-si 13488, Republic of Korea; myungjun.kim@lignex1.com; 5 Marine Domain & Security Research Department, Korea Institute of Ocean Science and Technology, Busan 49112, Republic of Korea; jidae@kiost.ac.kr

**Keywords:** unmanned surface vehicle, underwater thruster, fault diagnosis, vibration signal, continuous wavelet transform, transfer learning, time-frequency analysis, frequency response

## Abstract

Among unmanned surface vehicle (USV) components, underwater thrusters are pivotal in their mission execution integrity. Yet, these thrusters directly interact with marine environments, making them perpetually susceptible to malfunctions. To diagnose thruster faults, a non-invasive and cost-effective vibration-based methodology that does not require altering existing systems is employed. However, the vibration data collected within the hull is influenced by propeller-fluid interactions, hull damping, and structural resonant frequencies, resulting in noise and unpredictability. Furthermore, to differentiate faults not only at fixed rotational speeds but also over the entire range of a thruster’s rotational speeds, traditional frequency analysis based on the Fourier transform cannot be utilized. Hence, Continuous Wavelet Transform (CWT), known for attributions encapsulating physical characteristics in both time-frequency domain nuances, was applied to address these complications and transform vibration data into a scalogram. CWT results are diagnosed using a Vision Transformer (ViT) classifier known for its global context awareness in image processing. The effectiveness of this diagnosis approach was verified through experiments using a USV designed for field experiments. Seven cases with different fault types and severity were diagnosed and yielded average accuracy of 0.9855 and 0.9908 at different vibration points, respectively.

## 1. Introduction

In maritime environments, systems are more susceptible to faults than in other environments due to environmental perturbations [1,2], corrosive conditions [3,4], and floating debris [5,6,7]. Moreover, recent research on navigational efficiency [8,9], use of renewable energy [10,11,12,13,14], situation awareness [11,15], improved communication methods [16,17], etc., applied to surface platforms is broadening spatial and temporal boundaries for unmanned surface platforms. Hence, with the anticipated expansion of unmanned vessels’ operational scope in the near future, if a vessel becomes immobilized due to a malfunction in the middle of the ocean, the time and cost for retrieval can be substantial. Therefore, it is imperative to undertake research to automate the condition monitoring and fault detection that were previously carried out by onboard ship engineers.

For conventional ships, maintenance regulations are well organized. A daily checkup on sensor values and checklist confirmation is routinely performed. Also, periodic maintenance, overhauls, and regulations of necessary replacement parts on board enable a ship to maintain long-term operation and maintenance [18]. However, most Unmanned Surface Vehicles (USVs) follow scheduled maintenance and reactive strategies, and most fault-related research is heavily focused on commercial-class surface vessels [19,20,21,22].

Within the components constituting USVs, the integrity of the propulsion system is a decisive factor for the successful completion of missions. Yet, owing to its direct interaction with the marine environment, this system is inherently susceptible to a broad spectrum of failures, prominently including sudden disruptions induced by external factors. The capability to efficiently identify and categorize these faults during maritime operations is crucial, as it enables the formulation of adaptive strategies in alignment with the system’s level of autonomy and redundant thrust capabilities [23]. Given these considerations, this research aims to diagnose the types and magnitude of underwater thruster faults of USVs.

However, if the system is not fabricated with consideration for fault diagnosis, it is challenging to measure parameters such as rotational speed, power consumption, and voltage in real-time without making alterations to the system. These parameters, which are directly connected to the vehicle’s control and propulsion, are difficult to apply as diagnostic parameters without prior integration in the system design. Additionally, for smaller vehicles not subject to the mandates of the Automatic Identification System (AIS), the lack of suitable Global Navigation Satellite System (GNSS) equipment poses an obstacle to the effective real-time measurement and utilization of dynamic state data [24].

Therefore, this study aims to employ vibration-based fault diagnosis as a non-invasive and sensitive method that allows the acquisition of high-quality data in real-time without any alterations to the system or internal wiring. Vibration sensors offer a cost-efficient alternative for acquiring diagnostic features when compared to other parameters such as power consumption, supply voltage, propeller blade rotation speed, and high-accuracy GNSS sentences. Fault diagnosis based on frequency analysis of vibration data has been frequently utilized in various fields due to its simplicity and the ability to observe the characteristics of different frequency components associated with faults [25,26]. The most commonly used thrust source for surface vehicles, the underwater thruster, is a type of rotating equipment that operates on rotational motion. Thus, the use of vibration-based fault diagnosis becomes straightforward in the ease of identifying the fundamental frequency (first-order vibration, 1X component), which occurs at the same speed/frequency as the rotor [27] (p. 42).

While the direct attachment of vibration sensors to thrusters facilitates the acquisition of characteristic data conducive to fault diagnosis, implementing sensors within the hull’s interior is a more pragmatic strategy for general applications. The hull’s interior comprises structures with disparate stiffness and mass, leading to a complex array of resonance frequencies and harmonics [27,28] (pp. 275–279). This complexity makes the collected data notably noisy and challenging to analyze. Hence, under these conditions, the identification and isolation of faults with varying thruster speeds pose an intricate problem [29]. Nevertheless, data for this study was acquired according to a driving profile that entailed a ten-second acceleration from a static state to maximum rotational speed, followed by a subsequent ten-second deceleration phase. This driving profile was used not just to facilitate the diagnosis of faults across the entire spectrum of the thruster’s possible rotational speed outcomes but also to enable a variation in the spectrum of frequencies applied for fault detection. This approach enhances the capability of structural frequency analysis, allowing for the examination of not only the thruster but also the joint condition or damage of other structures, thereby extending its functional scope beyond diagnosing faults at fixed, specific speeds [29,30] (pp. 249–259).

To address the inherent challenges in fault diagnosis via hull vibrations and to diagnose faults at varying rotational speeds of USVs’ propulsion systems, the Continuous Wavelet Transform (CWT) was applied to convert time-series vibration data into scalograms [30,31]. Although a number of methodologies have been applied for fault diagnosis at a constant rotation speed of USV thrusters [32,33,34], analyzing varying rotational speeds demands a methodology that simultaneously accounts for attributes in both the temporal and frequency domains. CWT, renowned for its ability to encapsulate both the physical characteristics and time-frequency domain nuances, has been extensively researched and validated within the realm of Physics-Informed Neural Networks (PINNs) [35]. Hence, scalogram images obtained as an output of CWT were applied as an input for deep neural network (DNN) classifiers. Leveraging the widely applied and validated DNN techniques in image classification through transfer learning. A particular emphasis was placed on the application of the Vision Transformer (ViT) model, which is known for its ability to increase global context awareness and transfer learning efficiency [36]. This paper also proposes to present the critical considerations and analytical reflections required during experimental procedures involving varying thruster rotational speeds and the subsequent application of a DNN classifier. A USV with wireless DAQ (Data Acquisition) and a remote-control system was fabricated to experiment with the viability of the approach. The experimentation was conducted at sea, encompassing the collection of both normal status data and data under simulated fault cases. Common fault scenarios that are attributed to external factors were selected as fault cases [32,37,38,39]. The experimental results of this study aim to demonstrate the proposed method’s effectiveness as a non-invasive technique capable of not only identifying the type but also the extent of the faults. The study illustrated some results of wavelet transformations conducted on vibrations transmitted to the interior of a hull in marine environments and analyzed the physical characteristics across the time-frequency domain. The study quantitatively evaluates the acuity of a wavelet-based DNN classifier and discusses potential avenues for future research.

## 2. Related Works and Backgrounds

This chapter examines fault diagnosis research, focusing on underwater thrusters and unmanned maritime platforms. In addition, this chapter intends to provide an overview of previous research conducted on the platforms analogous to the ones used in this study and supplement the conceptual basis of the vibration-based approaches employed in this research.

### 2.1. Related Works

#### 2.1.1. Fault-Related Research on Unmanned Marine Platforms

**Figure 1:**
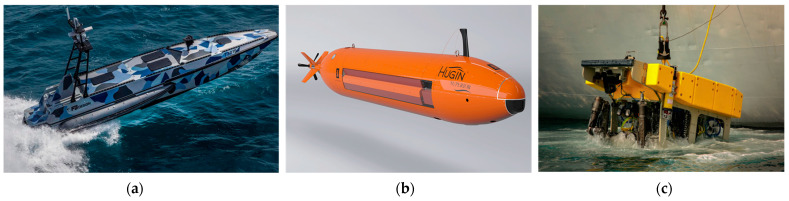
Types of unmanned marine platforms: (**a**) USV; (**b**) AUV; and (**c**) ROV.

Unmanned marine platforms can be categorized into various types, with three primary groups depicted in Figure 1. USVs navigating the water’s surface, Autonomous Underwater Vehicles (AUVs) functioning wirelessly, and Remotely Operated Vehicles (ROVs) that operate underwater tethered by cables. Due to its unique operating methods, areas of activity, and structural differences, each platform requires different methodologies and approaches for fault detection and diagnosis.

ROVs commonly adopt an over-actuated system (more actuators than the minimum required for controlling all its degrees of freedom) for enhanced maneuverability, stability in various underwater tasks, and increased payload capabilities. Their design allows for power supply via an umbilical cable, affording greater flexibility in the number of thrusters utilized compared to other marine platforms and offering the substantial benefit of ease in recovery in the event of a malfunction. Consequently, there has been a vigorous pursuit of research in fault tolerance using remaining thrusters in cases of individual thruster failures [23,39,40].

AUVs, particularly those designed as torpedo-type, are generally non-over-actuated. Even AUVs with specialized designs and objectives are conservatively designed in terms of thruster quantity due to the limitations of continuous energy supply for prolonged operations. As wireless platforms, AUVs encounter communication challenges with operators, leading to active research in risk analysis and reliability, focusing not only on fault diagnosis but also on sustained operational stability [39,41].

In contrast, USVs, unlike their submergible counterparts, benefit from the feasibility of real-time positioning via GNSS and radio communications, making their retrieval comparatively straightforward. Hence, research in this domain has primarily focused on navigation and guidance rather than fault diagnosis-related issues involving field testing [9,42,43]. However, as recent technological advancements extend the spatio-temporal capabilities of USVs, they will also increase difficulties associated with retrieval distance and cost. As such, automated condition monitoring, fault detection, and prognosis will become compelling areas for research.

#### 2.1.2. Underwater Thruster Stand-Alone Fault Detection and Diagnosis Research

Numerous studies have been reported on detecting and diagnosing underwater thruster faults and their stand-alone experiments. Fault diagnosis was performed using a small underwater thruster’s current and underwater acoustic data as features, employing a 1D-CNN (Convolutional Neural Network)-based deep learning structure and visualization through t-SNE (T-distributed Stochastic Neighbor Embedding) [33]. A thruster model was driven based on the control input, rotation speed, and current of the thruster, and the residual between the thruster model and actual data was used as a feature to compare classification performance through an MLP (Multi-Layer Perceptron) and LSTM (Long Short-Term Memory)-based classifier [44]. A fault diagnosis method was proposed using both current-rotational speed correlation analysis and support vector machines [45]. A test bed and data collection system for thruster experimentation were used and research was conducted on fault diagnosis using underwater acoustic data. Clustering and frequency analysis-based methods were used to identify fault features [34].

This study aims to diagnose faults through signals from vibration sensors attached to the hull. Hence, this research requires a method of vibration-based fault diagnosis that is sensitive and capable of handling noise, taking into account the structural influence and the damping effect of the fluid-contacting bottom surface. Hence, USV, which is capable of DAQ and field tests, was introduced to acquire actual noisy vibration data.

### 2.2. Backgrounds

**Figure 2:**
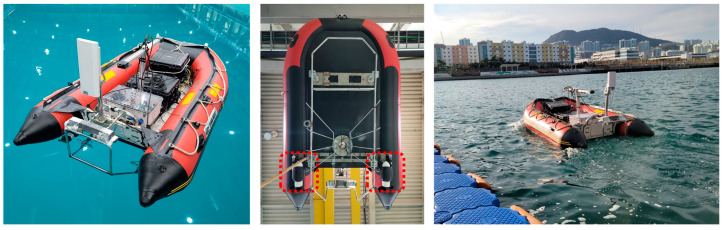
Overview of FDD USV.

This section provides an overview of previous research on the platform analogous to the one used in this study and supplements the conceptual basis of the vibration-based approaches employed in this research. The platform is designed for Fault Detection and Diagnosis USV (FDD USV) and has been revised for different research purposes. The FDD USV used in a series of previous studies and for this research is shown in Figure 2.

In prior studies involving FDD USV, extensive analyses were conducted on data parameters such as rotational speed, power consumption, supply voltage, and vibration to determine thruster malfunctions due to external causes. It was determined through experimental validation that, among these parameters, vibration presented the highest degree of sensitivity for fault identification [46]. Further research in FDD USVs also involved the application of dimension reduction and entropy-based numerical transformation techniques for each fault case [47]. The significance of vibration data was not only underscored in the context of FDD USV’s previous works but also broader applications involving the fault diagnosis of various rotational machines [26]. When factors such as propeller blade damage, temporary obstructions, or biological fouling occur, they cause a shift in the center of gravity away from the geometric center aligned with the propeller blade’s rotational axis. This shift results in a misalignment of the geometric center, leading to an imbalance in the rotating body [46]. This rotational imbalance acts as a vibromotive force, which is then transmitted through the structural components of the hull and detected by vibration sensors mounted inside.

The previous experiments with FDD USV were conducted in calm and controlled environments like engineering water tanks, focusing on results under consistent control inputs (rotational speeds). These conditions ensured isolation from external marine disturbances such as wind or waves, allowing fault identification within specific rotational speed ranges. In contrast to certain land-based rotational machinery, USVs frequently encounter scenarios where their rotational speeds are subject to continual variations during operation. Consequently, this research incorporates a fault diagnosis methodology that effectively copes with environmental perturbations and adapts to environments with fluctuating rotational speeds. This is achieved through the application of CWT and ViT techniques, demonstrating an advanced approach suitable for the dynamic operating conditions of USV.

## 3. Methods

This chapter provides a detailed explanation of the components that make up the workflow to achieve the objectives of this study, as well as an overview of the entire workflow process.

### 3.1. Fault-Induced Vibromotive Force

**Figure 3:**
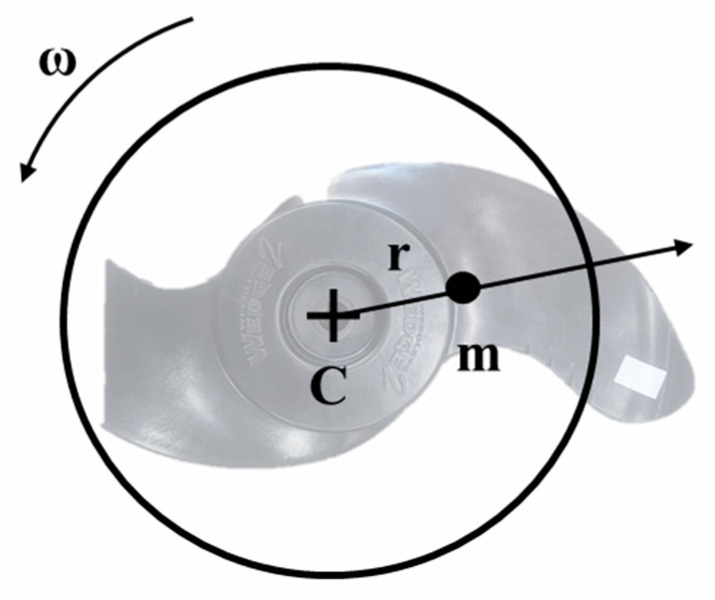
Imbalance on the underwater thruster blade.

The study explicitly targets faults like propeller blade damage and debris entanglement. Such faults cause a shift in the center of mass of the thruster blades, leading to rotational unbalance (rotor imbalance) and vibration. The concept of rotor imbalance is illustrated in Figure 3, with formulas representing the centrifugal force due to mass displacement written in Equation (1) and the resulting vibrations detailed in Equation (2).

(1) $$F_{c}=mr\omega^{2}$$

(2) $$V=\frac{F_{c}}{k_{str}}$$

**Figure 4:**
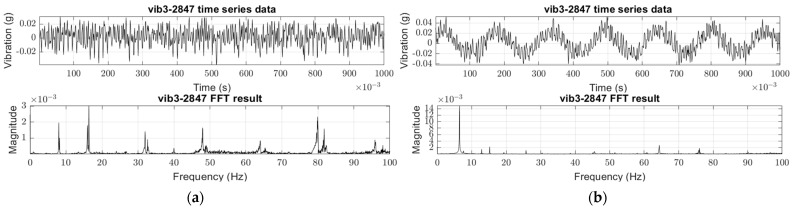
Sample vibration data and its Fast Fourier Transform result (FFT): (**a**) Normal (healthy) thruster raw vibration and 1X component; (**b**) Faulty thruster (breakage; 21% of diameter) raw vibration and 1X component.

In the formula, $F_{c}$ represents the centrifugal force caused by imbalance, r is the distance between the geometric center and the shifted center of mass, ω denotes angular velocity, and m indicates the imbalance mass resulting from damage or entanglement, referring specifically to the weight of a blade in case of blade breakage. V Signifies the vibration caused by imbalance, while $k_{str}$ represents the structure’s dynamic stiffness. The magnitude of vibration caused by rotor imbalance is proportional to the imbalance mass and the square of the angular velocity [46]. The vibration frequency corresponds to the rotational speed of the body, denoted as the first-order vibration, fundamental vibration, 1X component, etc. In a normal thruster, the rotor imbalance is minimal, leading to a smaller magnitude of the first-order vibration. However, in the event of entanglement or relevant faults, the first-order vibration’s magnitude increases. An illustration of the difference between normal conditions and fault conditions is shown in Figure 4. The figure illustrates the difference between normal and faulty thruster raw vibration data and their first-order vibration. Following the onset of a fault, the vibration signal reveals an enhanced periodicity that aligns with the blade’s rotational speed. The Fourier transform outcomes further substantiate the amplification of the first-order vibration, indicating a direct correlation between the fault condition and the specific vibrational pattern observed.

### 3.2. Wavelets and Continuous Wavelet Transform

Wavelet is a short-lived wavelike oscillation localized in time. To be defined as a wavelet, two essential conditions must be satisfied [30]

1. Admissibility condition

As shown in Equation (3), it means that the wavelet has no zero-frequency component or that the wavelet ψ(t) must have a zero mean.

(3) $$\int_{-\infty }^{\infty }\psi \left(t\right)dt=0$$

Interestingly, sinusoidal functions, which are fundamental elements of the Fourier transform and serve a contrasting purpose, also have a zero mean over the entire interval. The second condition that distinguishes sinusoidal functions from wavelets is shown in Equation (4).

2 Finite energy

The wavelet ψ(t) must have zero energy.

(4) $$\int_{-\infty }^{\infty }|\psi \left(t\right){|}^{2}dt<\infty$$

Having finite energy, wavelets possess the property of being localized in time. This is in contrast to Fourier transforms, which decompose infinite sinusoidal functions. Because wavelets exist within a localized time frame, they can capture information in both time and frequency domains. This study employs the CWT, a wavelet application, to generate scalogram images. The mathematical representation of CWT is formulated as Equation (5). This method is integral to the research’s objective of analyzing data across both time and frequency dimensions, providing a comprehensive analysis encompassing physical characteristics caused by faults.

(5) $$T\left(s,\tau \right)=\int_{-\infty }^{\infty }f\left(t\right)\cdot \psi_{s,\tau }^{*}dt=\langle f\left(t\right),\psi_{s,\tau }^{\;}\rangle$$

Here, f(t) is the signal of interest to be transformed and $\psi_{s,\tau }^{*}$ is a complex conjugate of the wavelet transform. Wavelet $\psi_{s,\tau }$ scaled (s) and translated (τ) over time, which can be represented as shown in Equation (4). Adjustments in the scale parameter s, facilitate the acquisition of frequency data, while modifications in the translation parameter τ, enable the gathering of temporal domain information. Wavelet $\psi_{s,\tau }$ in Equation (6) is a normalized form without consideration of complex-part.

(6) $$\psi_{s,\tau }=\frac{1}{\sqrt{s}}\psi \left(\frac{t-\tau }{s}\right)$$

T(s,τ)  is a convolutional result between the signal of interest f(t) and $\psi_{s,\tau }$. This outcome reflects the degree to which the scaled and translated wavelet $\psi_{s,\tau }$ contributes to composing the signal. As such, T(s,τ) serves as an indicator of the correlation between the original signal *f*(*t*) and $\psi_{s,\tau }$, with higher values (attributes to the brightness in the scalogram) denoting more remarkable similarity and lower values (darkness in the scalogram) indicating lesser similarity. The generation of a scalogram image is achieved by varying the values of *s* and τ and plotting these convolution results in alignment with time-frequency space.

The choice of wavelets varies across applications, with this study employing the Morlet wavelet, known for its real part’s resemblance to a sinusoidal wave overlaid with a Gaussian distribution. This wavelet has been widely used in different fault diagnosis applications due to its similarity to mechanical vibration patterns [48]. The formulation of the Morlet wavelet is presented in Equation (7).

(7) $$\psi \left(t\right)=\pi^{-1/4}\left(e^{i2\pi f_{0}t}-e^{-{\left(2\pi f_{0}\right)}^{2}/2}\right)e^{-t^{2}/2}$$

**Figure 5:**
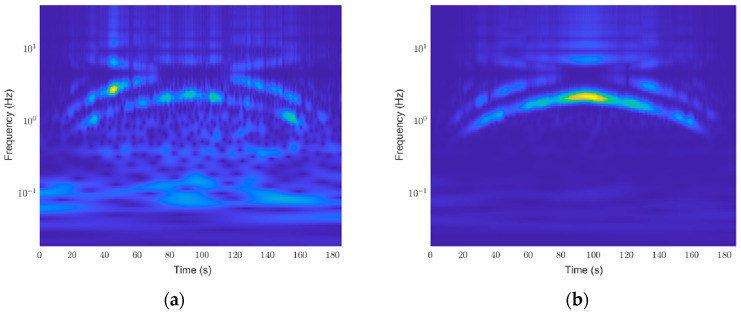
Sample scalogram results: (**a**) Normal (healthy) thruster scalogram; (**b**) Faulty thruster (breakage; 21% of diameter) scalogram.

An example of a scalogram generated from the results of the CWT is shown in Figure 5.

### 3.3. Vision Transformer (ViT)

**Figure 6:**
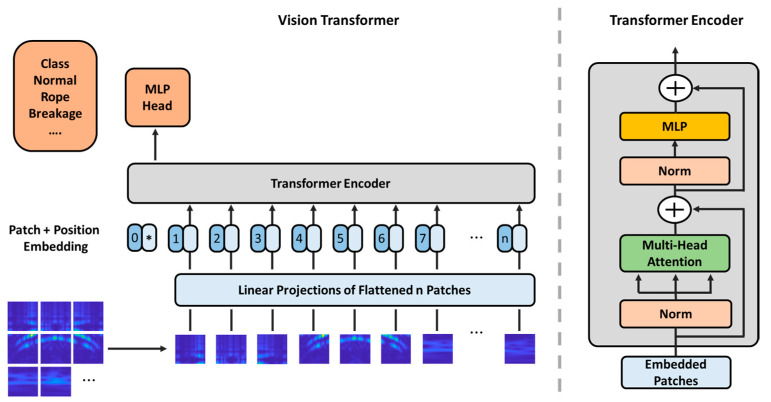
The ViT Architecture.

In the realm of image analysis, deep neural network (DNN) methodologies have been rigorously developed, enabling the utilization of pre-trained models across a broad spectrum of applications. Among these developments, the ViT, which utilizes transformer architecture: a structure initially emerging in natural language processing, has been adapted for vision-related tasks and is gaining traction in various disciplines. The ViT marks a departure from traditional CNN usage in vision tasks, instead leveraging the Transformer’s multi-head attention (self-attention) architecture to achieve significant performance. The ViT partitions an image into patches, processes each patch as a token, equivalent to a word in a sentence, and then employs a standard transformer architecture to process these tokens. The architectural framework of ViT is illustrated in Figure 6 [36]. Vibration scalogram datasets for both normal and various fault states from maritime experiments were stored according to their respective case labels. Then, the network’s head was then replaced with a new one tailored for the thruster fault diagnosis task, followed by fine-tuning on this new dataset.

**Figure 7:**
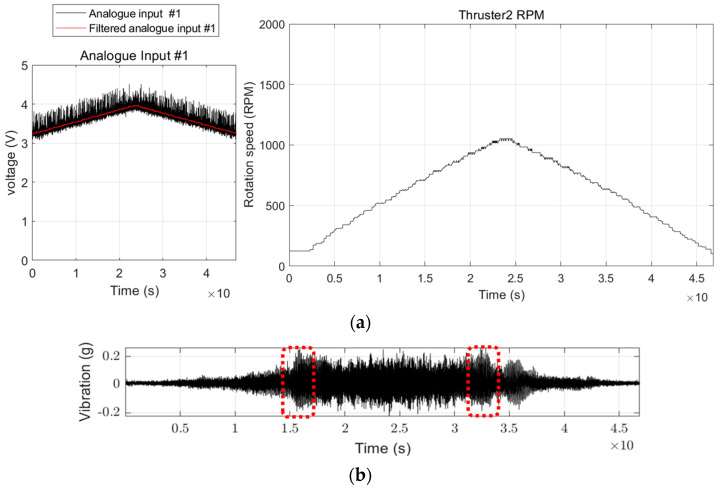
Sample data profile of FDD USV: (**a**) Analogue input, Rotational speed; (**b**) Acquired vibration data and FDD USV’s natural frequency (red dotted square).

In the ViT framework, the most distinctive aspect is segmenting images into patches and their subsequent arrangement into sequences, known as position embedding. This attributes ViT to various benefits, notably global context awareness and enhanced efficiency in transfer learning. ViT has been empirically validated to outperform many conventional CNN-based DNN classifiers under specific prerequisites [36,49]. Figure 7 depicts the dataset following a rotational speed-varying driving data profile intended for use in this research and sample data.

The driving profile entails an acceleration from a static state to maximum rotational speed, followed by a subsequent deceleration phase. As shown in Figure 7b, the magnitude of vibration does not increase in tandem with increasing rotational speed. Furthermore, the presence of the structure’s main natural frequency plays a significant role in analyzing the vibratory temporal response. Hence, ViT was selected as the foundational architecture for the image classifier in this study, attributing to its capacity to assimilate characteristic information relative to location.

### 3.4. Experiment Settings

**Figure 8:**
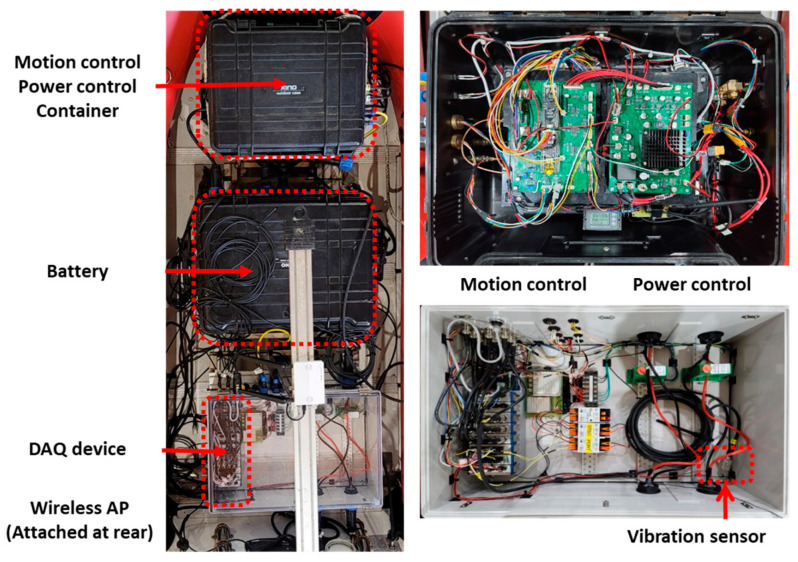
Hardware configuration of FDD USV control systems.

This research verified the methodology’s effectiveness through sea trials using a modified FDD USV capable of remote, real-time data acquisition. The control system configuration of the FDD USV used in this study is depicted in Figure 8.

**Figure 9:**
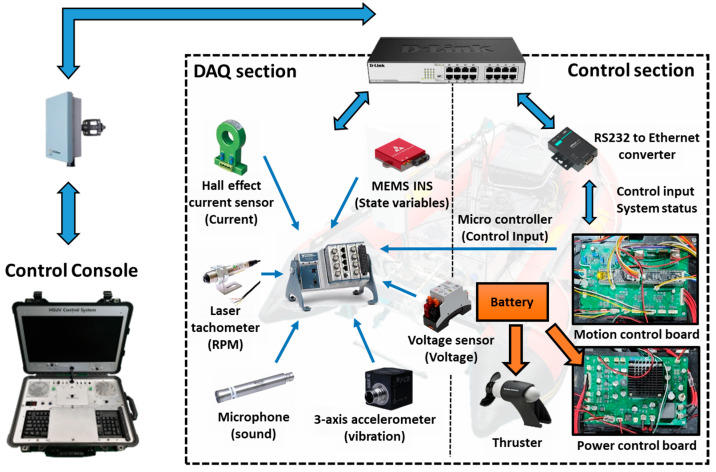
Configuration of FDD USV control and DAQ system.

The workflow of the remote control and the DAQ system through the FDD USV is illustrated in Figure 9.

The design was separated into two primary sections: the DAQ section for data collection and transmission and the control section for overall management. The control section incorporated a Motion control board with individual thruster control, path tracing control, dynamic positioning functions, and a power control board for managing various sensors and the device’s power distribution. The motion control board was integrated with the NI compact DAQ system and a microcontroller unit (MCU) to facilitate remote data acquisition at user-defined times and intervals.

The detailed experimental procedure is as follows. Initially, the power and communication status between the ground control console and the FDD USV are checked to ensure smooth operation. Then, the function of the thruster’s operational state and essential navigation performance are checked, including the status of the GNSS equipment used to track the platform’s position during experiments. Additionally, the operation of the rendezvous mode, which returns the platform to the launch position if the navigation control loop stops for any reason or if communication from the shore is interrupted for a certain designated period, is checked.

The DAQ process is primarily conducted from the onshore control console. The control console connects to the NI DAQ equipment’s IP address and control section IP address through an AP device that relays the connection between the shore and the FDD USV. The control section controls navigation systems and thrusters to execute the desired data profile. The NI DAQ equipment operates via the National Instruments’ FlexLogger 2021 R1 program and is connected to the control section using a hardware triggering method to repeatedly capture data at consistent intervals. This hardware triggering allows navigation software to execute control for the data profile while starting data logging. It stops the DAQ equipment’s logging function once control for the data profile is completed. The navigation program and thruster control software are programmed in C language on the control section’s Micro Controller Unit (MCU) and the GUI program on the onshore control console, which executes control commands developed in C#.

Additionally, to automate the acquisition of repetitive data and dataset creation, a macro program was developed in C# to automate the generation of data files through the FlexLogger program, execution of the NI DAQ equipment, thruster operation according to the data profile, stopping of DAQ equipment, and extraction of acquired data. The program to convert the acquired data into CSV format, preprocessing, and iterating the Continuous Wavelet Transform (CWT) was written in MATLAB R2023b. The program stores the data for each fault case in separate folders and automatically processes and performs CWT on the data within each folder, creating scalograms for each. As a result, separate folders containing only the scalogram images for each fault case are created. Finally, folders named after each fault case can be processed through a MATLAB-written ViT transfer learning program to obtain classification results.

**Figure 10:**
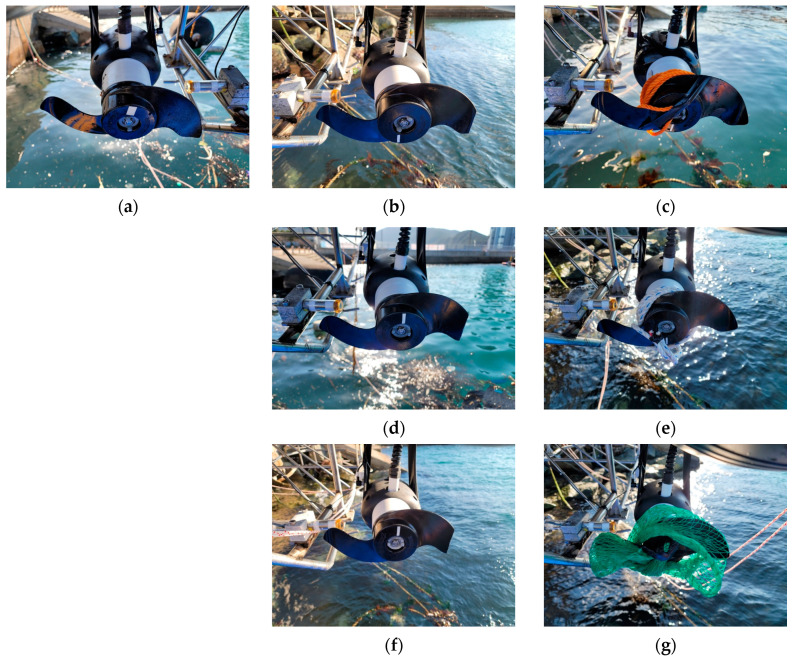
Test cases: (**a**) Normal(healthy); (**b**) Breakage 7%; (**c**) Thin rope entanglement; (**d**) Breakage 14%; (**e**) Rope entanglement; (**f**) Breakage 21%; and (**g**) Net entanglement.

**Figure 11:**
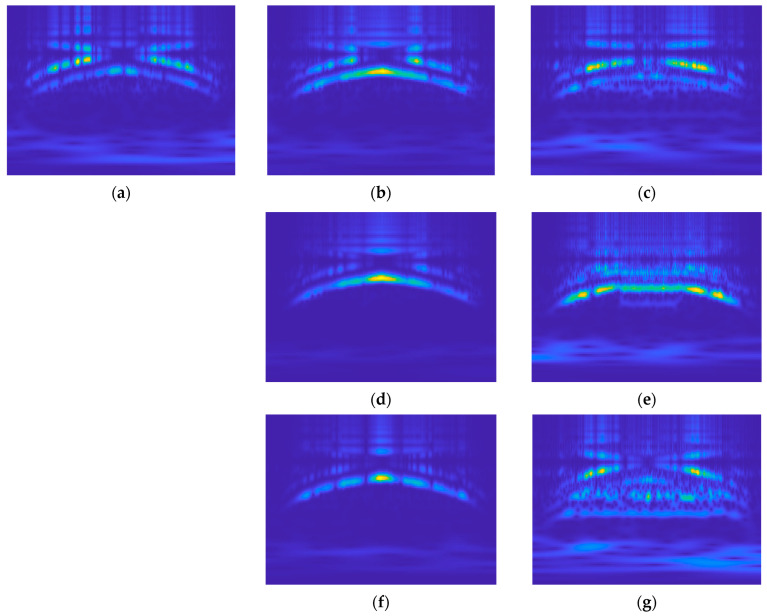
Sample result of CWT for test cases: (**a**) Normal (healthy); (**b**) Breakage 7%; (**c**) Thin rope entanglement; (**d**) Breakage 14%; (**e**) Rope entanglement; (**f**) Breakage 21%; (**g**) Net entanglement.

Specific faults, such as blade breakage and entanglements, typically resulting from external factors, were synthetically induced to simulate the dataset for the experiment. Simulated faults were applied to the starboard side thruster for all cases. The data acquisition was executed by a data profile comprising ten-second intervals of acceleration and deceleration each. Figure 10, in the study, provides a visual representation of both the normal and propeller fault cases utilized in these experiments. The results of the CWT corresponding to the sequence shown in Figure 10 are presented in Figure 11.

Figure 10b,d,f depict cases simulating breakage faults in propellers, with damages of 7%, 14%, and 21% relative to the radius of the propeller. Figure 10c,e,g represent entanglement-type faults, showcasing scenarios with a thin rope and a rope of different diameters entangled, as well as a net entanglement.

Figure 11 shows that all cases depict first-order vibration aligning with the rotational speed variations in the data profile, yet breakage cases and entanglement cases have discernable differences. In scenarios involving breakage, a pronounced enhancement of the first-order vibration is evident. In contrast, during entanglement events, the first-order vibration remains detectable; however, characteristics such as its inability to exceed a certain threshold due to speed-induced overload become apparent. Additionally, entanglements involving turbulent interactions with water lead to a noticeable increase in residual vibration components, yielding distinct and insightful outcomes across varying circumstances. Still, distinguishing between the normal state shown in Figure 11a and the minor faults depicted in Figure 11b,c remains challenging. Furthermore, in the case of breakage, the emphasis on the first-order vibration makes it difficult to differentiate between them visually.

**Figure 12:**
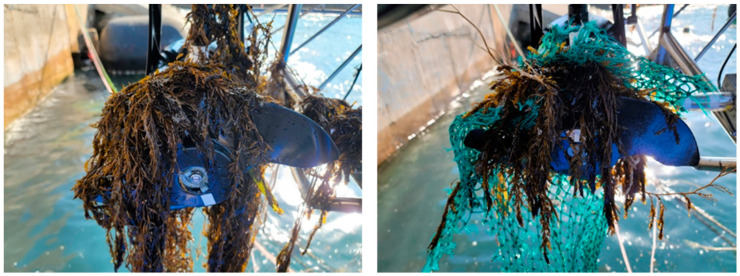
Naturally-occurred thruster entanglement faults during the experiment.

The study faced challenges in maintaining a uniform sample size across the datasets due to difficulty replicating severe faults, experimental disruptions caused by spontaneous naturally occurring faults, as shown in Figure 12, and adverse weather conditions. The dataset sizes for each case are listed in Table 1.

**Figure 13:**
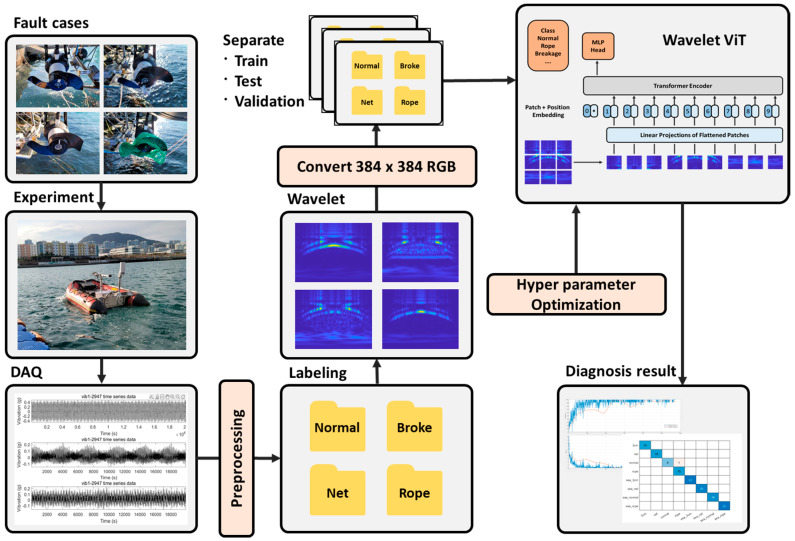
Overall fault diagnosis framework.

Each dataset outlined in Table 1 was converted into scalogram images and subsequently transformed into RGB images of dimensions 384 × 384 × 3, aligning with the ViT’s input layer specifications. These images were segregated into training, testing, and validation sets for targeted applications within the ViT framework. Utilizing a pre-trained ViT model, adaptations were made to the input and output layers to align with the transfer learning objectives of this research. Figure 13 illustrates the comprehensive framework for fault diagnosis, as designed to achieve the objectives of this research.

## 4. Results and Discussion

**Figure 14:**
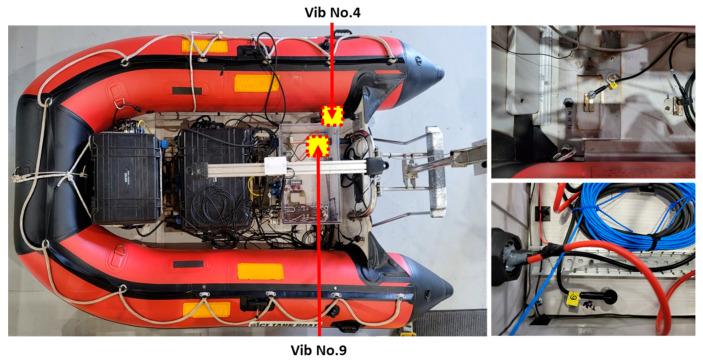
Vibration sensor attachment position of both vib No.4 and vib No.9.

The analysis of the results will focus on the classification outcomes and accuracy of the wavelet and Vision Transformer (ViT), as well as the confusion matrix. The study concentrated on the results from two sensors located inside the hull. The attachment positions of these two sensors are depicted in Figure 14. One sensor, labeled as vib No.4, was affixed upper deck, near the structure for fixing the starboard side thruster that simulated faults. The other sensor, vib No.9, was attached inside the container housing the DAQ system.

Only the vibration data from sensors vib No.4 and vib No.9 were selectively stored from the seven types of maritime experiment data. After transforming these data into scalograms, they were used to train a wavelet ViT classifier. The execution and transfer learning process of the ViT was conducted through MATLAB 2023b. The base model used for transfer learning was the “base-16-imagenet-384” from MATLAB, with 86.8 million learnable parameters. The adjustable parameters (hyperparameters) of the wavelet ViT classifier model utilized in this research are shown in Table 2.

**Figure 15:**
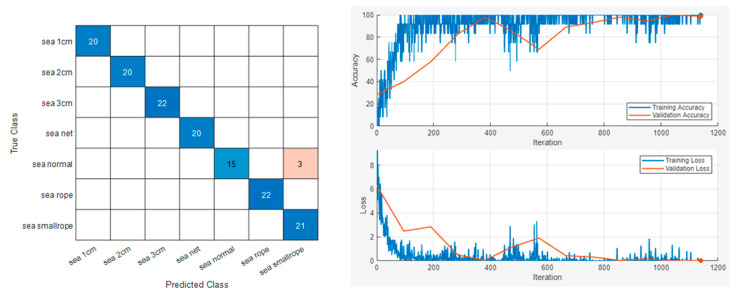
Vib No.4 maritime experiment dataset sample classification result.

**Figure 16:**
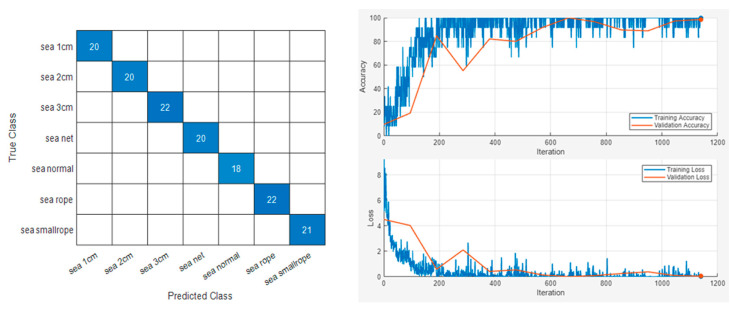
Vib No.9 maritime experiment dataset sample classification result.

The sample results of this classification are displayed in Figure 15 and Figure 16. Repeated results for each model are shown in Table 3.

The result demonstrated its capability of identifying minor faults, such as 7% breakage and thin rope entanglement, which were previously challenging to discern using other FDD USV-related research with different sensors and methodologies. This illustrates the classification methodology’s efficacy across various operational conditions.

**Figure 17:**
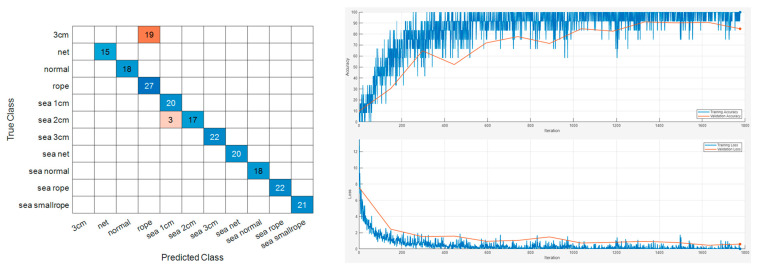
Vib No.4 maritime and on-ground experiment dataset classification result.

**Figure 18:**
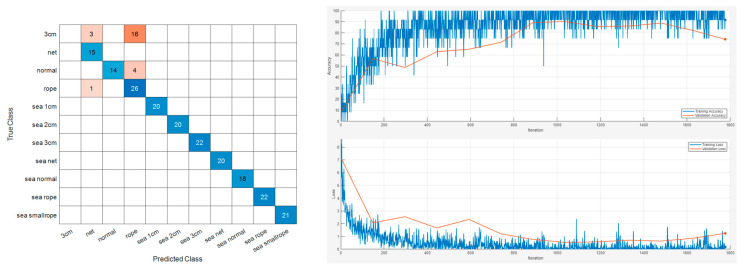
Vib No.9 maritime and on-ground experiment dataset classification result.

**Figure A1:**
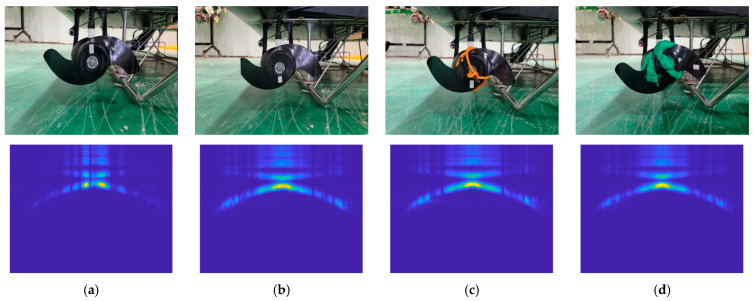
On-ground collected fault case and its resulting CWT result: (**a**) Normal; (**b**) Breakage 21%; (**c**) Thin rope entanglement; (**d**) Net entanglement.

The study aimed to classify maritime data but also incorporated more challenging on-ground experiment data into the training of the same model. The test settings and sample scalogram results are depicted in Appendix A, Figure A1. The sample results of this extended analysis, which includes both maritime and on-ground data, are presented in Figure 17 and Figure 18. Repeated results for each model are shown in Table 4.

The integration of on-ground and maritime data for fault diagnosis resulted in a noticeable decrease in overall accuracy. Given that the primary objective of this research is the diagnosis of faults within maritime data, a detailed examination of this phenomenon has been provided in Appendix A.

An interesting discussion point is that despite requiring significantly more training time under similar hyperparameter conditions, the ViT did not show a noticeable difference in thruster scalogram classifying accuracy compared to traditional CNN-based classifiers like ResNet50 or VGG counterparts. In an effort to explore this matter, repeated training of both CNN and ViT models was considered, but it was beyond the scope of this paper and challenging to discern whether the lack of significant performance difference was due to dataset size or the inherent complexity of the task.

During the training of the wavelet ViT model with maritime and on-ground datasets, it was observed that training accuracy tends to decrease when the number of epochs exceeds 14, likely due to dataset insufficiency and resultant overfitting issues. Similar to considerations regarding comparisons with CNN models for USV thruster diagnostics, further research seems necessary upon acquiring more data. Future studies will also aim to publish data not only from various vibration sensors but also include thruster-specific current consumption, voltage, noise, and USV states (position, velocity, and acceleration) after augmenting the dataset.

However, the study observed sensitive responses from the wavelet-based learning method to faults in marine platform thrusters and confirmed that ViT effectively distinguishes scalograms depicting the time-frequency distribution of vibrations transmitted inside the hull from underwater thrusters. Expanding this research into anomaly detection, which requires fewer additional datasets, could lead to more general and practical studies.

## 5. Conclusions

The underwater thrusters of USVs are crucial for mission execution yet, due to their direct engagement with the marine environment, rendering them susceptible to faults. A non-invasive and cost-effective vibration-based methodology that does not require altering existing systems is employed to diagnose thruster faults and to identify faults across all rotational speeds of the thrusters, not just at stationary speeds.

This research focused on diagnosing thruster faults due to environmental factors, explicitly targeting thruster blade breakage and debris entanglement. These issues lead to a shift in the center of mass, resulting in rotational imbalance and subsequent vibrations. The study highlighted the challenge of signal attenuation and noise introduction as vibrations traverse through varied materials to the hull. A data profile was employed to address the dynamic range of rotational speeds, underscoring the limitations of conventional Fourier-based vibration analysis methods in diagnosing faults within such fluctuating frequency data.

Therefore, this study applied wavelet transform to acquire insights into the distribution of frequency components aligned with variations in rotational speed and temporal changes. The CWT, a specific wavelet technique, was utilized to transfigure one-dimensional vibrational time-series data into scalogram representations. These scalogram images were then diagnosed for normal and various fault conditions using a ViT-based classifier. To empirically substantiate the efficacy of the proposed methodology, a custom USV (FDD USV) was fabricated for experimental trials at sea, conducting maritime experiments and facilitating the direct examination of vibrational data attributes and scalogram configurations under varied operational and fault-induced scenarios.

The FDD USV was engineered for both maritime navigation and wireless acquisition of large-scale data to a land-based control console. The research includes acquiring experimental data across seven maritime fault scenarios, including normal operations, by following a specific data profile of accelerating and decelerating the thruster from stationary to maximum rotation and back within 10 s. Data was captured at a 100 Hz sampling rate, and scalogram sample images for each fault condition were analyzed. The classification was performed using the proposed wavelet-ViT method on vibration data collected from two points inside the hull. Sensors affixed to the upper deck of the hull demonstrated an average accuracy of 0.9855, whereas those installed within a DAQ-equipped container achieved an average accuracy of 0.9905. Furthermore, classification efforts extended to eleven scenarios, incorporating four datasets from terrestrial tests, where sensors on the hull’s deck and within the container reported average accuracies of 0.9009 and 0.8761, respectively, illustrating the classification methodology’s efficacy across various operational conditions.

## Figures and Tables

**Table 1:** Number of acquired datasets for all cases.

Type	Normal	Breakage 7%	Breakage 14%	Breakage 21%
Number of data	185	200	200	220
Type	Thin rope entanglement	Rope Entanglement	Net entanglement	
Number of data	210	218	203	
Type *	Normal *	Breakage 21% *	Thin rope entanglement *	Net entanglement *
Number of data	191	194	269	150

* On-ground experiment datasets. Figures are shown in Appendix A, Figure A1.

**Table 2:** The adjustable parameters of the ViT classifier.

Parameters	Value
Input image size	[384, 384, 3]
Number of Transformer blocks	12
Number of attention heads	12
Position encoding dimension	2D
Optimizer	Adam (learning rate: $1\times 10^{-3}$)
Layer	143
Patch size	16
Batch size	12
Max epoch	12
Dropout probability	0.1
Attention dropout probability	0.1

**Table 3:** Accuracies of the maritime experiment classification result.

Sensor Number	Accuracy
Vib No.4	0.9790
Vib No.4	0.9840
Vib No.4	0.9790
Vib No.4	1
Vib No.9	1
Vib No.9	1
Vib No.9	0.9790
Vib No.9	0.9840

**Table 4:** Accuracies of maritime and on-ground experiment classification result.

Sensor Number	Accuracy
Vib No.4	0.9009
Vib No.4	0.9054
Vib No.4	0.9009
Vib No.4	0.8964
Vib No.9	0.8919
Vib No.9	0.8649
Vib No.9	0.8873
Vib No.9	0.8603

## Data Availability

Data are contained within the article.

## Appendix A

The comparative analysis of the CWT results from the on-ground and ocean experiments shows significant distinctions. In instances of imbalance-induced faults, there is a notable amplification of the primary component (first order, identical with rotor speed)’s impact. It is postulated that in on-ground experiments, this prominence of the primary component can be attributed to the absence of damping and interactions caused by fluids. This leads to the hypothesis that the observed effects are primarily mechanical in nature, occurring without the complicating influences of fluid dynamics. However, the CWT findings from the ocean experiment, while identifying changes in the first order vibration, also showed the presence of additional elements in other regions. This variation is hypothesized to stem from residual vibrations due to fluid interaction, especially pronounced in entanglement cases where the contact area with the fluid is enlarged, leading to the detection of multiple components beyond the first order.
